# Rheology as a Tool to Predict the Release of Alpha-Lipoic Acid from Emulsions Used for the Prevention of Skin Aging

**DOI:** 10.1155/2015/818656

**Published:** 2015-12-16

**Authors:** Vera Lucia Borges Isaac, Bruna Galdorfini Chiari-Andréo, Joana Marques Marto, Jemima Daniela Dias Moraes, Beatriz Alves Leone, Marcos Antonio Corrêa, Helena Margarida Ribeiro

**Affiliations:** ^1^Faculdade de Ciências Farmacêuticas, UNESP - Univ Estadual Paulista, LaCos, Rodovia Araraquara Jau, Km 1, CEP 14800-850 Araraquara, SP, Brazil; ^2^Centro Universitário de Araraquara (UNIARA), Rua Carlos Gomes 1338, 14801-340 Araraquara, SP, Brazil; ^3^Research Institute for Medicines and Pharmaceutical Sciences (iMed.UL), Faculty of Pharmacy, University of Lisbon, Avenida Prof. Gama Pinto, 1649-003 Lisbon, Portugal

## Abstract

The availability of an active substance through the skin depends basically on two consecutive steps: the release of this substance from the vehicle and its subsequent permeation through the skin. Hence, studies on the specific properties of vehicles, such as their rheological behavior, are of great interest in the field of dermatological products. Recent studies have shown the influence of the rheological features of a vehicle on the release of drugs and active compounds from the formulation. In this context, the aim of this study was to evaluate the influence of the rheological features of two different emulsion formulations on the release of alpha-lipoic acid. Alpha-lipoic acid (ALA) was chosen for this study because of its antioxidant characteristics, which could be useful for the prevention of skin diseases and aging. The rheological and mechanical behavior and the* in vitro* release profile were assayed. The results showed that rheological features, such as viscosity, thixotropy, and compliance, strongly influenced the release of ALA from the emulsion and that the presence of a hydrophilic polymer in one of the emulsions was an important factor affecting the rheology and, therefore, the release of ALA.

## 1. Introduction

Many factors are involved in the percutaneous absorption process and a number of studies have been performed in the search for alternatives and ways of improving this process, including research about the use of permeation enhancers in topical formulations and the development of new drug delivery systems [1–7].

The release characteristics of a drug from a dermatological vehicle can be assessed by studying the* in vitro* release profile, which provides very useful data [8, 9]. Thus, during the development of dermatological products, it is adequate to employ* in vitro* release tests to select vehicles that can provide reasonable therapeutic activity.

An important barrier to the percutaneous absorption process is the protective layers of the* stratum corneum* whose composition results in many drugs being prevented from crossing this barrier and reaching their site of action [10–12].

However, to be effective, a topical formulation must be able to allow the release of the active compound(s) or drug, and this ingredient needs to have the ability of penetrating the skin at suitable concentrations to exert its biological activity [13, 14].

Active compounds incorporated in unsuitable vehicles may penetrate the skin little or not at all, so the vehicle is a limiting factor, influencing the pharmaceutical performance. Studies have shown that the rheological properties of vehicles can influence the release profile of drugs, being a crucial step for the penetration of the skin by the active substance [15–18]. Also, other physical-chemical characteristics have been studied aiming at elucidating the interferences of them in the process of drug release, such as the surface free energy [19].

This subject was investigated earlier by Barry (1983) [13] who criticized several scientific studies for not considering the correlation between the viscosity of a preparation and the rate of drug release because, according to the author, the rheological characteristics of a product often affect the release of active compounds.

Over the last decade, research has afforded increasing importance to alpha-lipoic acid (1,2-dithiolane-3-pentanoic acid) as a powerful antioxidant, effective in scavenging free radicals, including reactive oxygen species, such as superoxide ions, hydroxyl radicals, peroxyl radicals, and singlet oxygen [20]. Alpha-lipoic acid (ALA) is a sulfhydryl compound found naturally in virtually all plant and animal species and in both prokaryotic and eukaryotic cells [21]. In the human body, it is bonded to lysine residues and acts as a cofactor in various multienzyme complexes [22].

Nevertheless, there is often little or no free ALA in tissues [23], so a topical antioxidant formulation containing this natural antioxidant could be used to protect the skin against the effects of ultraviolet rays, such as photoaging and skin cancer [24].

To investigate the influence of the rheological behavior of two different emulsions on the release of active substances from them, alpha-lipoic acid (ALA) was employed as a model compound in this study.

In light of the above statements, the aim of this study was to correlate the rheological behavior and the release profile of two different emulsions containing ALA (see Supplementary Material available online at http://dx.doi.org/10.1155/2015/818656).

## 2. Materials and Methods

### 2.1. Preparation of Emulsions

Two O/W emulsions (A and B) (Table 1) were prepared. The oily phase (Phase A) and the aqueous phase (Phase B) were heated to 75 ± 2°C separately. The aqueous phase was poured into the oily phase with continuous stirring until cooling.

The ALA, at 2%, was dispersed separately in the propylene glycol (Phase C) used in each formulation. This solution was then poured into the fresh emulsion while still hot and mixed well to achieve a completely homogeneous emulsion.

### 2.2. Structure Analysis of Emulsions

#### 2.2.1. Droplet Size Analysis of Emulsions

The size distribution of the emulsions was measured by light scattering using a Malvern Mastersizer 2000 (Malvern Instruments, Worcestershire, UK) coupled with a Hydro S accessory. For a correct turbidity, about 0.5 g of each formulation (A and B), corresponding to an obscuration between 10% and 20%, was added in the sample chamber containing 120 to 150 mL of water using a stirrer at 700 rpm. Data was expressed in terms of relative distribution of volume of particles and given as diameter values corresponding to percentiles of 10%, 50%, and 90% (mean ± SD; *n* = 6). The span value is a statistical parameter useful for characterizing the wideness of the particle size distribution (see the following equation):

(1) $$\begin{array}{c} \mathrm{Span}=\frac{d\left(90\right)-d\left(10\right)}{d\left(50\right)}. \end{array}$$

#### 2.2.2. Microscopy Analysis

A computerized image analysis device was used for the microscopic observations, connected to an Olympus BX51 microscope in bright field (Olympus, Japan). Samples were examined after preparation and storage at room temperature.

### 2.3. Study of Rheological Behavior

The rheological tests were performed with a Haake RS-1 Rheometer, using the cone-plate sensor (C35/2°Ti). In the first test, flow curves were generated by ramping the shear rate from 0 to 100 s^−1^ in 120 seconds (ascent curve) and then from 100 to 0 s^−1^ in 120 seconds (descent curve) and recording the shear stress throughout.

Next, the stress sweep test was conducted at a frequency of 1 Hz, with a range of shear stress from 0 to 50 Pa, and the frequency sweep test was performed over a frequency range from 0.01 to 10 Hz, at a shear stress of 1 Pa for emulsion A and 0.5 Pa for emulsion B. The creep and recovery test was carried out with a shear stress of 1 Pa for emulsion A and 0.5 Pa for emulsion B, allowing 300 seconds for creep and 300 seconds for relaxation.

All tests were performed on samples of about 1 g, at 32 ± 0.5°C. All experiments were performed in triplicate.

### 2.4. Surface Tension

Surface tension measurements of the emulsions using a Krüss Tensiometer K12 (Germany) at 25°C were performed. The Wilhelmy plate technique was applied in the Wilhelmy Constant Run programme mode. A platinum plate was partially immersed into the surface layer of an aqueous phase and the monitored surface tension decreased with time, while the plate remained into position. The analysis ceased when the surface tension value was stabilized. Prior to each analysis, the surface tension of bidistilled water (Strom) was measured as control.

### 2.5. *In Vitro* Release Assay

To assess the release profile of the formulations, Franz-type diffusion cells, with a release area of 1.77 cm^2^, were used. The receptor solution was sodium phosphate buffer (pH 7.4) containing 1% of polysorbate 80, which was maintained at 37 ± 2°C and stirred at 300 rpm throughout the experimental period. The diffusion cell was assembled with hydrophilic synthetic membranes of cellulose acetate, of pore size 0.45 *μ*m (Sigma-Aldrich, USA), separating the two compartments. Approximately 270 mg of emulsion A or B, containing 2% alpha-lipoic acid, was spread evenly over the membrane. A base emulsion of each, without ALA, was used as the blank. Samples were collected from the receptor solution after 2, 4, and 8 hours and analyzed by UV spectrophotometry at 334 nm.

An analytical curve was constructed with a certified reference solution of 2 mg/mL of alpha-lipoic acid in the receptor medium. This stock solution was diluted in the same medium to obtain solutions at concentrations of 1.4, 1.2, 1.0, 0.8, 0.6, 0.4, and 0.2 mg/mL, which were analysed in the UV spectrophotometer (Hitachi U-2001) at 334 nm.

Release data were fitted to zero-order, first-order, and Higuchi kinetic models.

### 2.6. Statistical Analysis

All analysis was performed at least in triplicate; average values and standard deviations were calculated. ANOVA and Tukey's test were used to differentiate release data. Differences were considered to be significant when *p* < 0.05.

## 3. Results and Discussion

### 3.1. Structure Analysis of Emulsions

#### 3.1.1. Droplet Size Analysis of Emulsions

**Figure 1:**
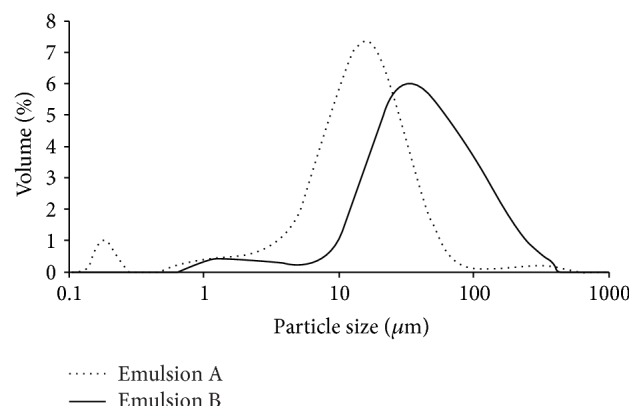
Droplet size distribution of emulsions A and B storage at room temperature.

The presence of carbomer ingredient influences the droplet size distribution. Both emulsions present a bimodal population (Figure 1). Nevertheless, the droplet size (90% of the droplets) immediately after preparation is different for both emulsions (40.07 ± 2.76 *μ*m and 145.48 ± 16.76 *μ*m for emulsions A and B, resp.) (Table 2). Emulsion B seems to present a higher droplet size dispersion which was translated by a higher standard deviation. For bimodal dispersions of droplets it is important to know the composition of the two populations. If the volume fractions of particles are the same, the viscosity of the bimodal dispersion can be lower than that of the monomodal system even when a decrease in the particle size occurs. However, according to Pal (1996) [25], the particle-size effect on rheological measurements is particularly important for emulsions with particles smaller than 1 *μ*m, which is the case of emulsion A (Figure 1). Nonetheless, in general, the reduction in droplet size results in an increase in the viscosity and storage modulus of the emulsions, which suggested an enhancement in emulsion stability.

#### 3.1.2. Microscopy Analysis

The light microscopy images revealed that the size of the droplets and the microstructure of the systems depended on the emulsifiers and emollients used.

**Figure 2:**
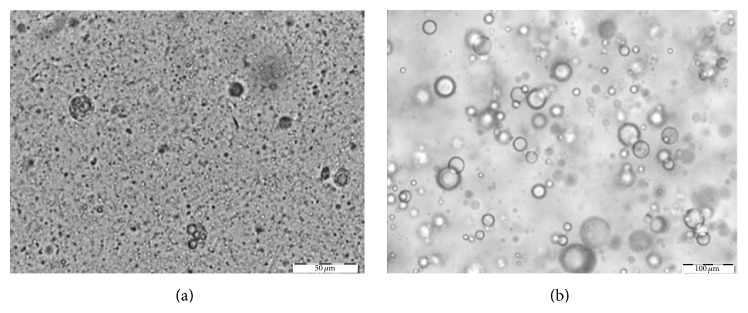
Photomicrographs of emulsions A (a) and B (b) (scale bar = 50 *μ*m).

In emulsion A, several small inner drops of oil were observed in the water phase. The droplets presented a smaller and nonhomogeneous size (Figure 2). In emulsion B, greater inner oil droplets with a nonhomogeneous size were seen.

Concerning the carbomer-based formulation, the results showed a significant influence on the microstructure of the emulsions and the microscopy analysis is in accordance with droplet size distribution. Figure 2 proved that ingredients such as polymers can modify the microstructure of the emulsion by, probably, raising the viscosity of the continuous phase or by causing adhesion between droplets without coalescence.

### 3.2. Study of the Rheological Profile

Rheology could be used as a tool to assess parameters that help in the evaluation of release of active compounds from vehicles.

In a review study, Barry (1983) [13] cited several scientific studies, criticizing their neglect of the correlation between the viscosity of a preparation and its release because, according to the author, the rheological characteristics of a product can affect the release of the active ingredient. Despite the importance of determining the rheological properties of a semisolid, such as a gel, studies rarely correlated parameters such as viscosity with release data [13]. In that study, the author correlated plastic viscosity with the diffusion of active ingredients from agar gel and found that the viscosity was inversely proportional to the rate of release. In another study by Marriott (1996) [26], the rate of absorption of active agents through the skin was inversely proportional to the viscosity of the vehicle.

Flow curves, stress and frequency sweeps, and creep and recovery assays were carried out to understand the rheological behavior of the emulsions.

**Figure 3:**
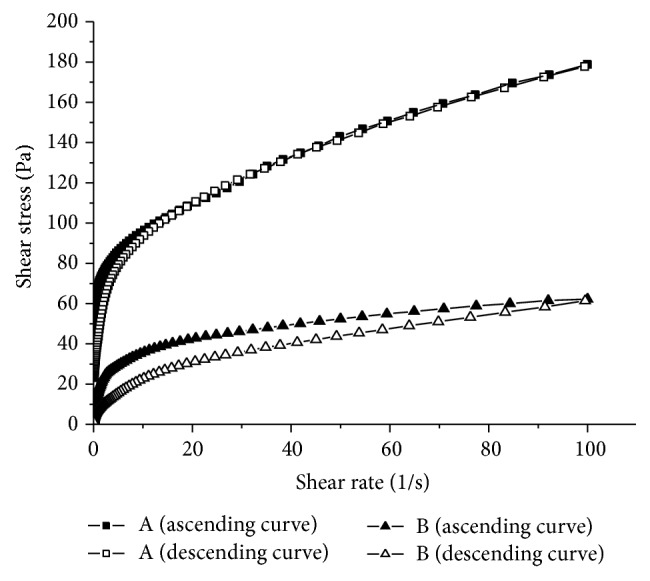
Flow curves of emulsions A and B.

The flow curves (Figure 3) showed that the emulsions were non-Newtonian fluids, which means that their viscosity varies as the shear rate varies. This characteristic, the nonlinear relation between the shear stress and the shear rate, is typical of non-Newtonian behavior [27].

The emulsions were also characterized as thixotropic, exhibiting a decrease in viscosity with increasing shear that is not completely recovered when the shear rate is ceased. It can be clearly seen that the hysteresis area of emulsion B is much larger than that of emulsion A (Table 3). This could influence the release rate of ALA [16], owing to the time necessary for the emulsion to recover its viscosity and initial structure when the shear rate that is imposed on it falls. Thus, active substances incorporated in more thixotropic systems have more time to be released faster in the period of low viscosity caused by the spreading of the emulsion over the skin by the consumer.

As it is possible to see also in Figure 3, emulsion A needs a higher shear stress to flow than emulsion B, showing that emulsion A is more structured than emulsion B, since it needs more shear stress per unit of area to be disrupted and flow.

The flow curve data were fitted to the Herschel-Bulkley rheological model. This model was chosen because it is a general power-law equation for non-Newtonian fluids [28] and includes a value for the yield stress, observed in the cosmetic emulsion studied. According to Schramm (2006) [27], rheological curve fitting allows confidence intervals to be set around the standard regression coefficients instead of comparing the standard curves with a specific flow, and thus it can be decided whether the material tested is within or outside the specifications of the model in question.

The Herschel-Bulkley model furnished the rheological parameters of yield stress (*τ*_0_), behavior index (*n*), consistency index (*K*), and the correlation coefficient (*r*) for the model fitting. The correlation coefficient, which measures the proportion of total variation of the mean explained by the regression, was above 0.99 for all fitted curves, demonstrating that the model was highly appropriate.

**Figure 4:**
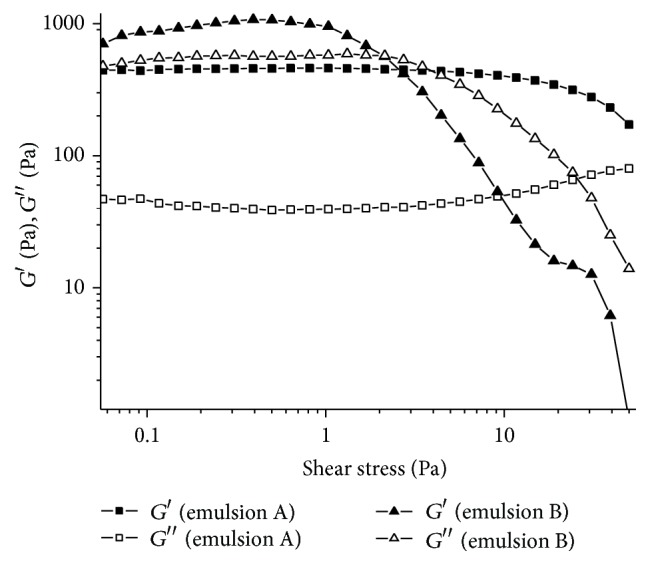
Shear stress sweeps of emulsions A and B.

The shear stress sweep precedes the frequency sweep and creep and recovery tests, making it possible to determine the values of shear stress, within a linear range, at which the sample does not suffer deformation (Figure 4), also called the linear viscoelastic region [29]. In the range from 0 to 5 Pa, sample A was not disrupted, while for sample B this was true in the range from 0 to 1.0 Pa. Thus, the values of *G*′ remained linear within this region of linear viscoelasticity, which can therefore indicate the suitable shear stress to be used in frequency sweep and creep and recovery tests.

Emulsion B exhibited higher *G*′ and *G*′′ values than emulsion A, but when subjected to increasing shear stress, B was disrupted (both moduli fell) earlier than emulsion A. This reflects how much the systems are structured, since the higher linear viscoelastic region is the higher microstructural stability of the sample is [30].

**Figure 5:**
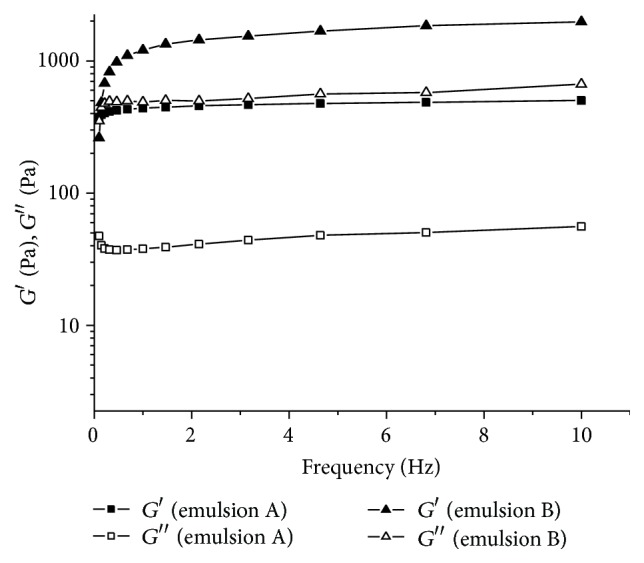
Frequency sweeps of emulsions A and B.

The frequency sweep curves of emulsion A (Figure 5) showed that in the range tested (1–10 Hz) there was practically no variation in the elastic and viscous moduli. Furthermore, viscoelastic behavior was seen over the whole range, since *G*′ (elastic modulus) was higher than *G*′′ (viscous modulus). The same relation is observed for emulsion B, but *G*′ and *G*′′ values for emulsion B are higher than for emulsion A.

When a viscoelastic material has a storage (or elastic) modulus higher than the viscous (or loss) modulus, the shear energy is temporarily stored during the test and can be retrieved later as usually occurs in O/W emulsion systems. Emulsion systems with this feature usually exhibit high stability.

**Figure 6:**
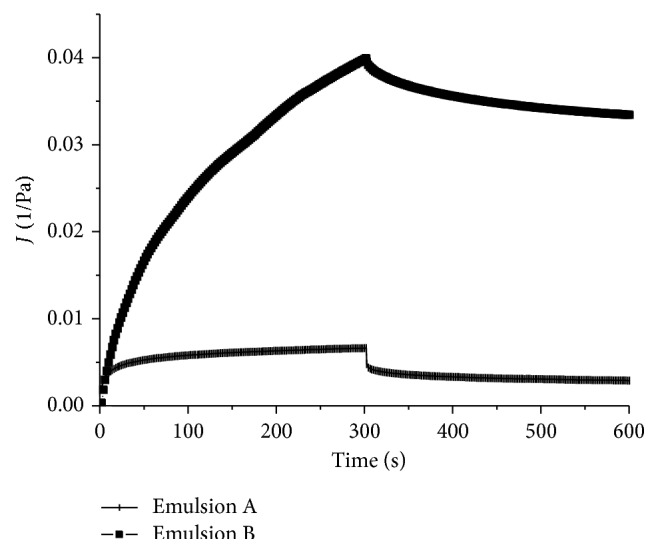
Creep and recovery of emulsions A and B (shear stress of 1 Pa for emulsion A and 0.5 Pa for emulsion B).

**Figure 7:**
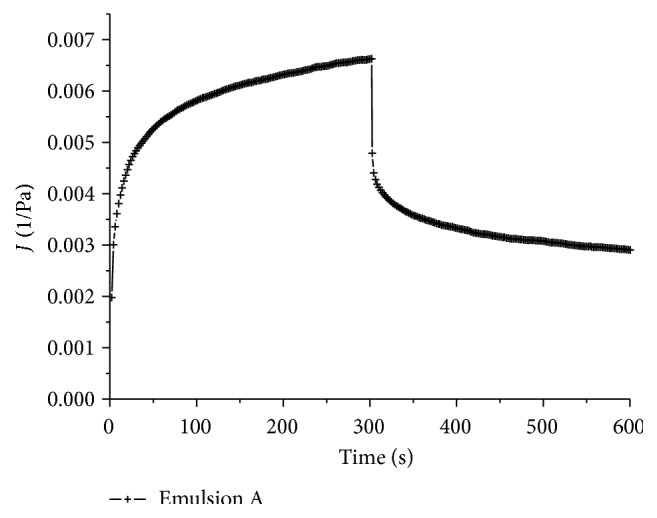
Creep and recovery of emulsion A (augmented scale).

When the emulsions were submitted to the predetermined shear stress (1 Pa for emulsion A and 0.5 Pa for emulsion B) for 300 seconds, in the creep and recovery test, samples A and B both suffered deformation, shown by the compliance value (*J*), but emulsion B was much more susceptible to this force (Figure 6). In the recovery part of this assay, when the shear stress was removed and the samples could recover their former structure, the elastic part of the deformation, more evident in emulsion A, was reversed (Figure 7). This response is quite common in polymer systems. In emulsion B, the recovery was almost negligible compared to emulsion A.

According to the rheological behavior data, comparing emulsions A and B, the first required a higher shear stress to be deformed than the second and when the forces acting on the emulsion ceased emulsion A recovered its structure faster. This could help predict the release profile of the two emulsions, with reference to the shear and time required by the emulsions to allow the release of ALA when applied to the skin. During spreading, emulsion B will “open” its structure more readily, allowing the ALA to be released more efficiently than emulsion A, apart from taking more time to recover its initial structure, providing more time for ALA diffusion. To verify this expectation, the release assay was performed.

### 3.3. Surface Tension

Surfactants are frequently used to stabilize an emulsion, decreasing the surface tension of multiphasic systems. Additionally, the measurement of surface tension could be a tool to understand the flow and the transport of the drugs across the skin [31].

The surface tensions of emulsions are shown in Table 4. The surface tension values for emulsion B are lower than those for emulsion A. In a recent study, Azarbayjani et al. (2010) [32] demonstrated that lower values for interfacial tension promoted the drug permeation and a similar result was obtained in this study (Table 5). Thus, both emulsions could improve the wetting properties and skin permeability. However, concerning emulsion A, this one exhibits higher surface tension than emulsion B, probably due to the fact that polymers are frequently added to increase the stability of an emulsion by thickening the external phase, increasing the viscosity of the external phase which plays a minor role for the stabilizing action. Thus, addition of a carbomer to the emulsion increases the interfacial tension of the multiphasic systems decreasing the wettability of active substance on the skin surface and a lower permeability of the drug, which is in accordance with* in vitro* release assay described below. Another factor that probably interferes in this parameter is the surfactants used, which was different in the two emulsions assessed.

### 3.4. *In Vitro* Release Assay

ALA in formulation A had a different release profile from that in formulation B (Table 5), possibly reflecting the great difference in rheological properties that were revealed during the rheological tests.

When the data were fitted to the zero-order, first-order, and Higuchi kinetic models by linear regression (Table 6), the regression coefficients (*R* ^2^) were calculated (Table 7).

For emulsion A, the results showed a release profile closest to the Higuchi model. Since this is best suited to controlled release systems, this indicates that the active substance release was controlled by diffusion. The Higuchi model describes the diffusion of substances from matrix systems, where the amount of substance released is proportional to the square root of time [33, 34].

For emulsion B, the most appropriate mathematical model was of zero order or first order, since the two models showed *R* ^2^ values close to 1. The zero-order model describes a system where the dissolution occurs at a constant rate, being independent of active substance concentration [35]. The first-order kinetic model is related to formulations of conventional and immediate release [36], with the amount released depending on the time of release and on the amount of active substance remaining in the formulation, so that the rate of release of the active substance falls [37]. The release of ALA from emulsion B was apparently immediate, since in the first 2 hours of assay 44.5% of the initial ALA was released, and the rate of release fell with time (Table 8). Although emulsion B released a larger amount of ALA than emulsion A, the rate of release from emulsion B was smaller than that from emulsion A. This occurred because of the reduction in the rate of release as the amount of ALA in the formulation decreased. According to this result, the first-order kinetic model is more suitable for emulsion B than the zero-order model.

The results obtained for emulsion A are consistent with the literature, since the presence of polymers in formulations slows and controls the release of the active substance. In a review paper by Lopes et al. (2005) [38], it was reported that the presence of water-soluble polymers, and especially hydroxypropyl methylcellulose (HPMC), slows the release of active substances from the vehicle. In a study by Monteiro et al. (2007) [34] it was proved that formulations containing, respectively, 30, 50, and 64 mg of HPMC released 88.82%, 75.43%, and 49.16% of the quercetin after 8 h of experiment, demonstrating that an increased proportion of HPMC delayed the release of quercetin.

From the above results, it is suggested that the rheological profile and the other physical-chemical characteristics of the two emulsions may have interfered significantly in the release of ALA by the vehicle, being possible to summarize that decreasing the viscosity, increasing the hysteresis area, increasing the droplet size, and decreasing the surface tension probably will be more effective in the release of the active substance from the vehicle. However, it should be done carefully, since many of these parameters also influence the system's stability.

A similar result was reported by Bruschi et al. (2007) [39] for formulations containing carbomer 934P with propolis extract. The authors observed that increasing the polymer concentration resulted in an increase in viscosity of the static system, which hindered with the release of the active substance. The same was observed here since emulsion A, containing carbomer dispersion, showed a release profile that is quite different from emulsion B, which lacks the polymer in its formulation.

The choice of a suitable system, the incorporation of an active substance, is of fundamental importance for the stability and availability of the active substance at the application site and, therefore, for its effectiveness. The vehicle has an influence on release, permeation, and retention of the active substance, taking a dominant role in dermatological therapy [35].

## 4. Conclusion

In conclusion, the release assay confirmed the prediction of release profile suggested by the rheological data, showing the influence of the rheological features on the release of active substances from the vehicles.

Thus, recalling that the permeation of an active substance through the skin depends basically on two consecutive steps, (1) the release of this substance from the vehicle and (2) its subsequent penetration through the skin, it is important to think about the first of these steps when developing a formulation for dermatological use. Therefore, it is crucial to consider carefully the ingredients of the vehicle and their effects on the rheological properties of it, since viscosity, thixotropy, compliance, and other rheological characteristics can exert a direct influence on the release of the active substance and hence on its action on the skin.

## Supplementary Material

Considering that the permeation of cosmetic active substances through the skin depends on the release of these substances from the vehicle, it is possible to understand that the rheological features of a cosmetic emulsion could influence in the permeation through the skin and, obviously, in the cosmetic effect of this substance. This research compares two formulations with different rheological characteristics through its release rate of alpha lipoic acid (ALA).

## Figures and Tables

**Table 1:** Qualitative and quantitative (%w/w) composition of emulsions A and B.

Component (INCI name)	A	B
Phase A		
Behenyl alcohol, Polyglyceryl-10 Pentastearate, Sodium stearoyl lactylate	4.00	—
Caprylic/capric triglyceride	2.00	—
*Octyl stearate*	1.00	—
Dibutyl adipate	4.00	—
Ceteareth-20	—	3.00
Cetearyl alcohol	—	3.00
Cetyl palmitate	—	3.00
*Isopropyl myristate*	—	4.00
PEG-75 lanolin	—	2.00
Dimethicone copolyol	—	1.00
Phase B		
BHT	0.10	0.10
Carbomer	2.00	—
EDTA	0.10	0.10
Methylparaben	0.18	0.18
Propylparaben	0.02	0.02
Metabisulfite	0.15	0.15
Triethanolamine	pH 6.00	pH 6.00
Water	80.45	77.45
Phase C		
Propylene glycol	4.00	4.00
Alpha-lipoic acid	2.00	2.00

**Table 2:** Droplet size distribution of emulsions A and B storage at room temperature, (*n* = 6, mean ± SD).

Emulsion	Span	*d*(10)	*d*(50)	*d*(90)
A	2.40 ± 0.09	3.36 ± 0.71	15.28 ± 0.80	40.07 ± 2.76
B	2.91 ± 0.28	14.31 ± 1.12	45.08 ± 3.00	145.48 ± 16.76

**Table 3:** Hysteresis areas of emulsions A and B (mean ± SD, *n* = 3).

Emulsion	Hysteresis area (Pa/s)
A	256.60 ± 10.74^a^
B	863.96 ± 40.20^b^

^a,b^Different letters mean statistically different values.

**Table 4:** Surface tension of emulsions A and B (mean ± SD, *n* = 3).

Emulsion	Surface tension (mN/m)
Water	70.76 ± 0.03
A	47.71 ± 0.86
B	36.43 ± 1.27

**Table 5:** ALA released from emulsions A and B in various periods of time (mean ± SD, *n* = 6).

	Time (h)	Amount of ALA (*μ*g/mL)	Amount of ALA per area (*μ*g/cm^2^)	% released
A	2	0 ± 0^a^	0	0
	4	292.42 ± 0.04^b^	1156.46	37.90
	8	366.13 ± 0.01^c^	1447.97	47.46

B	2	343.35 ± 0.01^a^	1357.88	44.50
	4	386.67 ± 0.01^b^	1529.20	50.12
	8	537.27 ± 0.01^c^	2124.79	69.64

Standard deviation (*n* = 6); SD <5% in all results.

^a,b,c^Different letters means statistically different values.

**Table 6:** Linear regression equations obtained in determining the reaction order of *in vitro* release of alpha-lipoic acid from formulations A and B.

Kinetic model	Emulsion A	Emulsion B
Zero-order	*y* = 217.26*x* − 145.75	*y* = 130.82*x* + 1060.08
First-order	*y* = 0.24*x* − 0.05	*y* = 0.03*x* + 1.57
Higuchi model	*y* = 723.98*x* − 579.82	*y* = 554*x* + 517.79

**Table 7:** Values of *R* ^2^ calculated to determine the best kinetic model for *in vitro* release of alpha-lipoic acid from formulations A and B.

Kinetic model	Emulsion A	Emulsion B
Zero-order	0.75	**0.98**
First-order	0.62	**0.99**
Higuchi model	**0.89**	0.95

**Table 8:** Rate constant (*k*) for release of alpha-lipoic acid from the formulations tested.

Emulsion	Rate constant (*k*) (*μ*g/mL/h)
A	61.02
B	32.32
