# Supplementary figures and images for: Rheology as a Tool to Predict the Release of Alpha-Lipoic Acid from Emulsions Used for the Prevention of Skin Aging
Source: Biomed Res Int. 2015 Dec 16;2015:818656. doi: 10.1155/2015/818656 (PMC4695648; doi:10.1155/2015/818656)

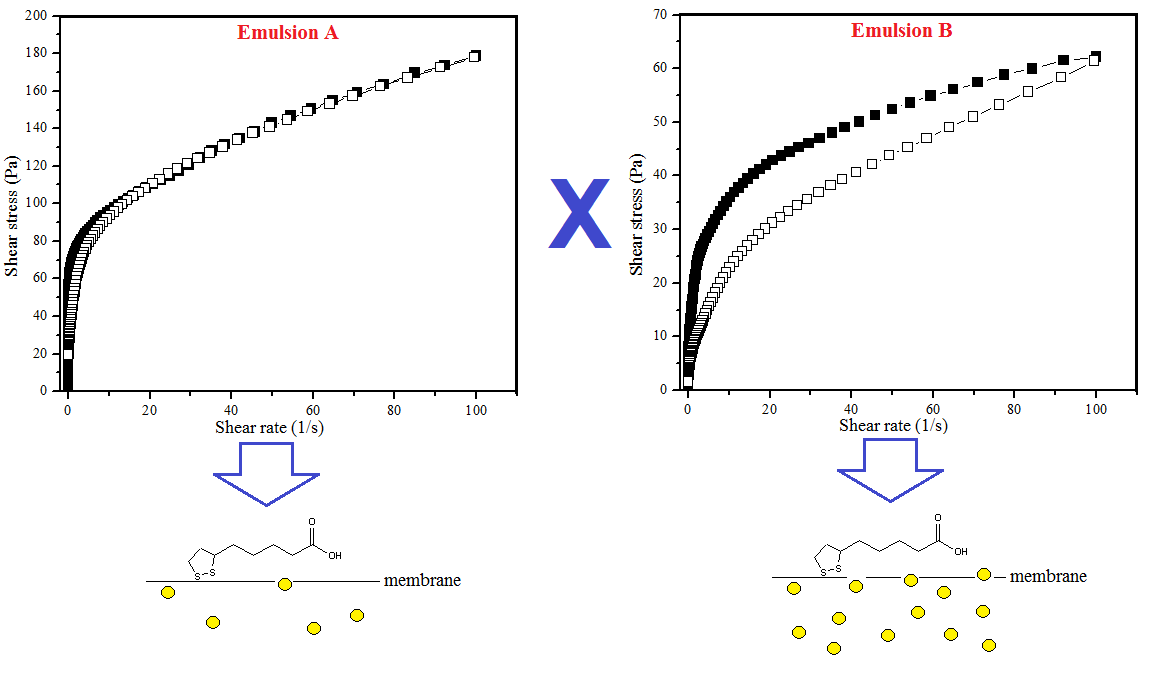

Supplement: Supplementary file 1 — Considering that the permeation of cosmetic active substances through the skin depends on the release of these substances from the vehicle, it is possible to understand that the rheological features of a cosmetic emulsion could influence in the permeation through the skin and, obviously, in the cosmetic effect of this substance. This research compares two formulations with different rheological characteristics through its release rate of alpha lipoic acid (ALA). [file 818656.f1.png]
